# Luminex and Other Multiplex High Throughput Technologies for the Identification of, and Host Response to, Environmental Triggers of Type 1 Diabetes

**DOI:** 10.1155/2015/326918

**Published:** 2015-03-25

**Authors:** Sharad Purohit, Ashok Sharma, Jin-Xiong She

**Affiliations:** ^1^Center for Biotechnology and Genomic Medicine (CBGM), Medical College of Georgia, Georgia Regents University, 1120 15th Street, Augusta, GA 30912, USA; ^2^Department of Pathology, Medical College of Georgia, Georgia Regents University, 1120 15th Street, Augusta, GA 30912, USA; ^3^Department of Biostatistics and Epidemiology, Medical College of Georgia, Georgia Regents University, 1120 15th Street, Augusta, GA 30912, USA

## Abstract

Complex interactions between a series of environmental factors and genes result in progression to clinical type 1 diabetes in genetically susceptible individuals. Despite several decades of research in the area, these interactions remain poorly understood. Several studies have yielded associations of certain foods, infections, and immunizations with the onset and progression of diabetes autoimmunity, but most findings are still inconclusive. Environmental triggers are difficult to identify mainly due to (i) large number and complex nature of environmental exposures, including bacteria, viruses, dietary factors, and environmental pollutants, (ii) reliance on low throughput technology, (iii) less efforts in quantifying host response, (iv) long silent period between the exposure and clinical onset of T1D which may lead to loss of the exposure fingerprints, and (v) limited sample sets. Recent development in multiplex technologies has enabled systematic evaluation of different classes of molecules or macroparticles in a high throughput manner. However, the use of multiplex assays in type 1 diabetes research is limited to cytokine assays. In this review, we will discuss the potential use of multiplex high throughput technologies in identification of environmental triggers and host response in type 1 diabetes.

## 1. Introduction

**Figure 1:**
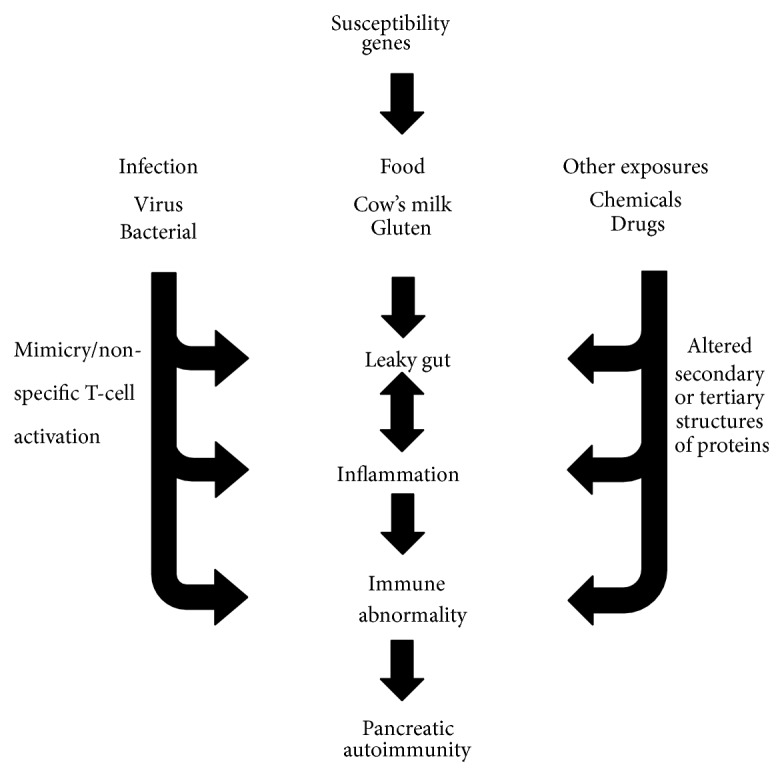
Susceptibility genes and environmental triggers in development and progression of type 1 diabetes. In genetically susceptible individuals, different classes of environmental exposures such as diet, infection, and pollutants lead to increase in peripheral and mucosal inflammation causing leaky gut and aberrant immune reaction towards pancreatic *β*-cells.

Type 1 diabetes (T1D) results from complex yet poorly defined interactions between environmental agents, the immune system, and genetic factors (Figure 1). T1D is a chronic T-cell mediated disease, characterized by selective loss of insulin-producing *β*-cells in the pancreatic islets [1]. There is an annual average of 3% increase in T1D incidence worldwide and the incidence rates are also increasing in the countries with no previous record of having T1D [2, 3]. It is believed that genetic susceptibility is a prerequisite for the development of T1D; however, not all genetically predisposed individuals develop clinical disease and subjects with low risk or protective genes also have been found to develop T1D. These observations suggest that apart from genetic susceptibility additional factors trigger the process of *β*-cell autoimmunity and subsequent clinical disease.

If these environmental triggers are known, change in life style is likely to offer the most powerful strategy for effective prevention of T1D. If successful, this approach can target the whole population or at least the population with increased genetic susceptibility. In pilot studies, dietary interventions have been successfully tested to manipulate appearance of *β*-cell autoimmunity in high risk children [4, 5]. However, there has been little progress in this area partly due to nonavailability of technologies to measure different types of environmental exposures and host response in large sample sets. The recently developed multiplex technologies have enabled the measurement of greater number of analytes in a high throughput manner.

## 2. Environmental Triggers of T1D

Several studies have observed seasonal patterns in the presence of serum antibody titers (incidence being more common during cold), in part due to the role of recent infections in the development of *β*-cell auto-antibodies [6]. Viral infections have been suggested to be responsible for T1D autoimmunity for a century, but recent studies have provided stronger data [7]. A number of viruses have been shown to be associated with T1D autoimmunity including* Enterovirus*, rubella, mumps, and rotavirus [8]. Taking into account the timing and profiles of the autoantibody peaks observed in several studies,* Enterovirus* infections appear to be the most probable trigger of *β*-cell autoimmunity [9]. Despite significant amount of evidence, the role of viruses in the development of T1D autoimmunity is not conclusive. The long silent period between the infection and clinical onset of T1D may lead to loss of the viral signatures in serum [9, 10]. Some authors believe that infections may protect from development of T1D autoimmunity. The proponents of the “hygiene hypothesis” suggest that children experiencing more infection in childhood are more protected; however there has been no consensus among researchers [11–13]. On the other hand the “trigger-booster hypothesis” claims that progression to clinical type 1 diabetes typically requires the unfortunate combination of genetic disease susceptibility, a diabetogenic trigger, and a high exposure to a driving antigen [14].

A number of dietary factors have also been found to be associated with development of T1D, including cow's milk, wheat gluten, and vitamin D deficiency [15, 16]. Some studies have shown the protective role of breast feeding and other nutrients [17, 18]. Several other studies have shown positive association of *β*-cell autoantibodies with introduction of milk based or wheat based formula early in life [19]. The results of these studies have always been mixed with no consensus on specific dietary factor or nutrient being conclusively responsible for development of T1D [20].

Recent studies suggest that *β*-cell autoantibodies are preceded by active inflammation [21]. Viral infections, dietary factors, and changes in gut microbiome lead to intestinal inflammation and may contribute to the increased permeability of the gut [16]. Vaarala et al. showed that the complex interactions between gut microbiome, intestinal permeability, and mucosal immunity contribute to the pathogenesis of T1D [22, 23]. These authors suggested that leaky gut allows entry to certain proteins present in cow's milk and wheat and as such leads to T1D autoimmunity in at risk subjects [22].

## 3. Measurement of Environmental Exposures and Host Response in T1D

Elucidation of the environmental exposure in T1D has been a highly contentious issue. Although studies have postulated a role of several environmental agents in T1D, progress in this area has been slow. This at least in part is attributed to the complex nature of the environmental exposures. A large number of environmental exposures need to be explored from different classes, including bacteria, viruses, dietary factors, and pollutants and the measurement involves different classes of molecules including DNA, RNA, proteins, metabolites, small molecules, and antibodies.

Also, due to a huge lag time between the time of exposure and the onset of disease, sometimes it is difficult to identify the environmental trigger itself. However, such an exposure may leave a signature or fingerprint which may be present for a longer time (host response). Thus measuring this host response may provide additional information correlated with the environmental triggers. For example, circulating levels of IgG and IgM against viruses have been shown in T1D patients [9, 24–26]. Similarly, measurement of cytokines, chemokines, and other plasma proteins could provide us a hint on the class of environmental exposures [27]. Measuring the host response may provide us unique fingerprints which may be used as additional markers for disease progression.

## 4. Current Approaches

Currently, many available “omics” technologies are being used to study the environmental exposures and host response in T1D. To identify molecular and cellular signatures, we have measured several classes of biomolecules in our laboratory [28–32]. ELISA assays are being used for detection of* Saccharomyces cerevisiae* [33], wheat protein Glb-3 [34, 35], gluten [36], gliadin, cow milk proteins in T1D, and celiac disease [37]. Giongo et al. used pyrosequencing approach to show that the intestinal microbiome of the children progressing to clinical disease was less diverse than healthy children [38]. PCR based typing was utilized to identify the* Enterovirus* DNA circulating in the serum of newly diagnosed T1D patients [16, 39]. However, the studies focusing on measurement of single environmental agent provide a skewed view on the environmental exposure. This can be remedied by measuring several types of environmental agents and host response to obtain a fingerprint of overall environmental exposure in a high throughput manner.

## 5. Multiplex Technologies

To measure several hundreds of the environmental triggers and the host response in larger sample sets economically, high throughput technologies are needed [28–32]. ELISAs or radioimmunoassays have been the preferred technologies for the measurement of low abundance agents in the serum. Recently, multiplex assays have been developed from traditional ELISA assays with the purpose of measuring multiple analytes in the same sample at the same time. Multiplex assays are available in several different formats based on the utilization of flow cytometry, chemiluminescence, and array technology (Table 1). Compared with traditional ELISA, multiplex arrays have a number of advantages including (i) high throughput multiplex analysis, (ii) less sample volume requirements, (iii) efficiency in terms of time and cost, (iv) ability to evaluate the levels of given analyte in the context of multiple others, (v) ability to perform repeated measures of the multiplex panels in the same experimental assay conditions, and (vi) ability to reliably detect analytes across a broad dynamic range of concentrations [40].

**Figure 2:**
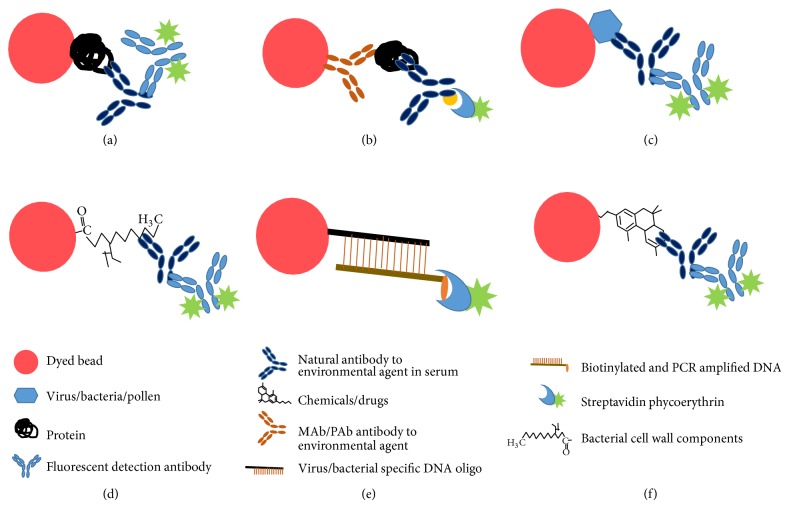
Luminex bead arrays could be used to detect different classes of environmental triggers. (a) Protein(s), (b) monoclonal (MAb) or polyclonal (PAb) antibodies, (c) viruses, (d) bacterial cell wall components, (e) DNA from virus/bacteria, and (f) chemicals/drugs can be covalently coupled to the beads. Coupled entities can be detected using fluorescently labeled appropriate detection agents.

Bead-based multiplex assays represent probably the most commonly used format developed by several companies. Multianalyte profiling (xMAP) technology from Luminex (http://www.luminexcorp.com/) and several other companies employ proprietary bead sets which are distinguishable under flow cytometry (Figure 2). The platform is a suspension array where capture moieties are covalently coupled with internally dyed microspheres, and phycoerythrin-labeled anti-human antibodies bind to the specific antigen-antibody complex on the bead set. Response is thus recognized and measured by the differences in both bead sets, with fluorogenic emissions detected using red (bead set) and green (detection of entities) lasers. The flexibility of the system allows covalent coupling and detection of several different classes of molecules or macroparticles.

## 6. Use of Multiplex Assays in Other Research Areas

Multiplex assays have been at the forefront for epidemic monitoring by health agencies in USA and abroad. Developments in the PCR technology and discrimination methods combined with the multiplex assays have improved the detection of coinfections with reduced cost and sample volumes required for analysis [40]. The most active research areas using the multiplex immunoassays are allergy, asthma, infectious disease, autoimmunity, and toxicology (Table 2). Extensive research efforts have been taken to test the feasibility of the Luminex xMAP technology to detect the autoantibodies to autoantigens, IgE response to grass and tree pollen, virus and bacterial serotypes, and weaponized microbial agents. Researchers in the fields of vaccine development and epidemiology have extensively documented the use of multiplexed assays to identify targets using antibody-based capture or DNA fragments specific to each serotype of bacteria or viruses. Using monoclonal antibodies to the individual serotypes, Yu et al. used Luminex technology to detect 26 different serotypes for* Streptococcus pneumoniae* in serum [41]. Other investigators have used antibody-based multiplex assays to characterize microbial pathogens and agents [42–44]. Conventional and real-time RT-PCR, combined with Luminex bead array, were used in detection of multiple viruses to identity the microbial agents in disease individuals [45, 46]. All these reports provide sufficient feasibility regarding the development of multiplex assays to identify the environmental triggers in T1D.

## 7. Technological Considerations for Developing Multiplex Assays for Environmental Triggers of T1D

Although multiplex technologies offer several advantages over ELISA approaches, caution must be exercised for developing assays. In this section some of the critical issues are discussed and possible solutions are offered for development of these assays.

1. Multiplex bead assays, by their very nature, involve measurement of several potential analytes in a single well. Therefore, cross-interactions between different capture antibodies and antigens in the sample/assay solution are inherently possible. Cross-reactivity of antibodies should be tested first and the lowest amount should be used to minimize such cross-reactions. The individual panels should be designed using the analytes with minimal cross-reactivity among the analytes and detection reagents. Secondly, multiplex assays are performed using a common binding and wash buffers, and these may not be the optimum conditions for all the analytes. This can be solved by creating custom multiplex assays having similar binding and washing conditions.
2. In the bead-based multiplex arrays, the reactions take place among capture entities and analytes which are freely mobile in solution, providing more sensitivity to measure circulating levels of analytes. However, abundant proteins present in bodily fluids, such as serum, may affect multiplex results. The abundant proteins serve both as reservoir and as carrier of small molecules such as cytokines and metabolites. The bound complexes may not get captured in the solution phase or the detecting reagent may not be able to access the required binding site. This interference from abundant proteins may require development of additional processing steps prior to multiplex assay.
3. The commercially available Luminex kits can measure up to 60 cytokines, chemokines, and ligands. However the linear range of the standard curve and the levels in the samples limits the number of analytes which can be measured simultaneously. This issue can be solved by performing pilot experiments to select the analytes having similar dynamic range (customized panels). For a particular value of sample dilution, the analyses will be selected in the same panel if their median fluorescence intensities fall in the linear range of the standard curve.
4. A defined set of principles are required to establish good laboratory practices and must be followed in the planning, performing, monitoring, recording, reporting, and archiving of all laboratory measurements. To prevent quality problems a good quality assurance policy must be established. The variations in high throughput measurements emerge from many sources. To reduce plate to plate variation and to produce consistent results over time, a dilution series of pooled control serum on each plate should be included for the normalization.
5. The bulk of our knowledge about T1D pathogenesis comes from studies of animal models. Data from human subjects are scarce and difficult to replicate for many reasons including, but not limited to, large variations of the studied phenotypes at the individual and population levels and differences in study design (insufficient sample size, poor matching of patients and controls, case/control versus prospective, and so forth). Important considerations in the proper design of human studies include prospective studies, which minimize many of the drawbacks associated with cross sectional comparisons that are commonly used in human studies.

## 8. Conclusions

Multiplex technologies offer opportunities to examine the different “classes” of environmental triggers of T1D in a time- and cost-efficient high throughput manner. While the use of such technologies is still at early stage, recent reports from other research areas highlight their usefulness and feasibility to evaluate the environmental exposure and host response in T1D pathogenesis. Also, multiplex technologies offer substantial sample savings over traditional ELISA measurements. Despite potential advantages of this new technology, expertise and experience are required for new assay development. We have used this technology in examining several classes of serum proteins in T1D [47–49]. In our view, multiplex technology could be successfully used for the evaluation of different classes of environmental exposures and host responses in T1D pathogenesis.

## Figures and Tables

**Table 1:** Characteristics of currently available array based high throughput technologies.

Technology/manufacturer	Maximum number of analytes	Maximum number of samples	Volume of sample	Dynamic range
Luminex/Luminexcorp	500	96 or 384	1–5 *µ*L	>4.5 logs
SimOa/Quanterix	10	384	1–10 *µ*L	>4 logs
Flow Cytomix/Afffymetrix	20	96	25 *µ*L	—
Cytometric Bead assay/BD biosciences	30	96	25–50 *µ*L	—
Barcoded Magnetic Beads/Applied Biocode	128	96	1–5 *µ*L	5 logs
Antibody arrays/Quansys Bioscience^*^	25	96	30–50 *µ*L	—
Antibody arrays/Meso Scale discovery^*^	10	96 or 384	30–50 *µ*L	>4 logs
Antigen Arrays/Thermo Scientific	10	96	40 *µ*L	—

^*^Detection is based on chemiluminescence or electrochemiluminescence.

**Table 2:** Research areas and available assays for Luminex platform.

Research Areas	Manufacturers	Species
Immunology/inflammation/apoptosis/tissue remodeling markers	Millipore, RnD Systems, Life Technologies, Luminex, Biorad	Hu/Ms/Rt/Ca/Mo
Phosphoproteins, signal transduction proteins	Millipore, Life Technologies, Biorad	Hu/MS
Cancer markers	Millipore, Biorad	Hu/Ms
Metabolic markers	Millipore, Life Technologies	Hu
Cardiovascular markers	Millipore, Life Technologies, Biorad	Hu
Toxicity markers	Millipore	Hu
Neuroscience	Millipore, Biorad	Hu
Antibody isotyping	Biorad	Hu/Ms
Auto-antibody measurement	One Lambda, Origene	Hu
Genotyping, epigenetics, and gene expression profiling	Affymetrix, One Lambda, Origene, Active Motiff	Hu
HLA typing	One Lambda	Hu
Environmental agents/allergens/food	Thermo Scientific	Hu
Bacterial/virus serotyping	Biovet/Luminex	Hu
Drugs/chemical	Under development in research laboratories	Hu
Vaccine testing	Luminex	Hu

Ca: cat, Hu: human, Mo: monkey, Ms: mouse, Rt: rat.
